# Capillary Blood Self-Sampling for Therapeutic Drug Monitoring: A Mixed-Methods Usability Study of the True Dose^®^ Kit

**DOI:** 10.1155/bmri/9973081

**Published:** 2025-07-09

**Authors:** Marie-Therése Crafoord, Elham Hedayati, Aina Johnsson

**Affiliations:** ^1^Department of Clinical Science and Education, Södersjukhuset (South Hospital), Karolinska Institutet, Stockholm, Sweden; ^2^Department of Oncology-Pathology, Karolinska Institutet, Stockholm, Sweden; ^3^Department of Oncology, Södersjukhuset (South Hospital), Stockholm, Sweden

**Keywords:** blood chemical analysis, blood specimen collection, clinical trials, drug approval, instructional film and video, user-centered design

## Abstract

**Objective:** This study evaluates the usability in accordance with EN 62366 of the True Dose kit for capillary blood self-sampling, to be used to analyze, specify, design, verify, and validate usability as it relates to the safety of medical devices focusing on user performance, experience, and user confidence through a mixed-methods approach. The usability engineering process is needed to fulfill user interface design requirements as part of product development at True Dose kit.

**Methods:** The study employed a convergent parallel design, integrating quantitative and qualitative data. Twenty-five participants, recruited through convenience sampling, used the True Dose kit to perform capillary blood self-sampling. Quantitative data were collected using three key performance metrics—collection of sufficient blood volume, absence of air bubbles, and duration of the self-sampling process—along with two questionnaires, the System Usability Scale (SUS) and the Test Feedback Questionnaire (TFQ), to comprehensively assess user performance and experience. In contrast, qualitative data were obtained from interviews and free-text responses. The integration of findings highlighted areas of convergence and divergence between the quantitative and qualitative data.

**Results:** The overall SUS score was 82.70, indicating high usability. However, participants reported challenges with the complexity of instructions and stress associated with blood flow management. Qualitative insights revealed a preference for simplified visual guidance and highlighted the need for precise feedback mechanisms to ensure the correct execution of sampling steps. Despite these challenges, 92% of participants collected the required blood sample, with a median self-sampling duration of 10.42 min.

**Conclusion:** The True Dose kit demonstrates high usability potential, but improvements in instructional design and stress management are needed to enhance user experience and ensure consistent performance.

**Trial Registration:** EUCT Number: 2024-514818-12-00

## 1. Introduction

Venipuncture is a cornerstone of phase 1 clinical trials and is critical in therapeutic drug monitoring (TDM) within clinical settings [1, 2]. This method is pivotal for pharmacokinetic analyses and is essential for understanding drug absorption, distribution, metabolism, and excretion. Additionally, TDM ensures that drug concentrations remain within therapeutic ranges, optimizing patient care while minimizing the risk of toxicity.

Traditionally, venipuncture has been regarded as the gold standard for blood sampling, particularly when volumes exceeding 1 mL are required [3–5]. However, this method has drawbacks, including its invasive nature, the need for a sterile environment, and the requirement for trained phlebotomists [6]. These limitations are further compounded by potential risks, such as pathogen exposure, the complexity of sample preparation, and the necessity for precise shipping conditions, all of which contribute to elevated healthcare costs [7].

The growing need for expanded population studies has highlighted the limitations of venipuncture. In particular, traditional blood collection methods need to be better suited for decentralized clinical trials or regular monitoring in routine clinical practice. The requirement for frequent visits to healthcare providers for blood sampling places a significant burden on patients, healthcare systems, and the pharmaceutical industry [8]. Vulnerable populations, such as immunocompromised individuals, face even significant risks, especially during pandemics.

Given the limitations of venipuncture, patient-centric microsampling techniques, which require less than 500 *μ*L of blood, offer a less invasive and more flexible alternative. Microsampling, especially capillary blood sampling, reduces the physical and logistical burdens associated with traditional blood sampling and TDM and enables more flexible and frequent monitoring, which is crucial for personalized treatment regimens [9–12]. Previous research has indicated that patients often consider at-home blood sampling devices to be more user-friendly than venipuncture, mainly due to shorter waiting times and the avoidance of crowded clinical environments [13, 14].

Several existing microsampling methods, such as dried blood spot (DBS) and volumetric absorptive microsampling (VAMS), have been explored for this purpose. However, these techniques are known to suffer from matrix effects, hematocrit bias, and preanalytical variability, which can compromise analytical accuracy and reproducibility [15–17]. To address these limitations, we developed the True Dose kit. This novel liquid-based capillary self-sampling technology incorporates two key innovations: (i) the integration of an internal standard (IS) directly within the collection tube at the point of sampling and (ii) immediate protein precipitation within the same tube [18]. Importantly, while plasma has traditionally been the standard matrix for TDM, it is subject to physiological fluctuations due to hydration status, posture, and food intake, particularly for lipophilic and protein-bound drugs [19, 20]. Moreover, plasma proteins like albumin and *α*1-acid glycoprotein, key players in drug binding, vary significantly among patients, whereas hemoglobin levels in whole blood remain relatively stable [19, 20]. Thus, measuring drug concentrations in whole blood can offer improved reliability, especially for cytotoxic agents like epirubicin, which have significant intracellular binding and redistribution. Moreover, this design eliminates the need for plasma separation or drying of blood samples, as well as manual IS and precipitation solution addition, vortexing, and subsequent sonication steps that are required in traditional venous blood collection and DBS or VAMS workflows.

Given these technological advances and the importance of usability in clinical workflows, usability is essential for adopting new medical technologies, as outlined in Nielsen's framework, which includes learnability, efficiency, memorability, error frequency, and user confidence [4, 21–23]. The ISO defines usability as specific users' effectiveness, efficiency, and confidence in achieving their goals in a particular context [24]. These principles are especially relevant for True Dose kit, a new technology designed for capillary blood self-sampling.

This study evaluates the usability of the True Dose kit using a mixed-methods approach, assessing user performance, experiences, and perceptions. The findings will inform kit refinement.

## 2. Concept

The main objective of the usability engineering process for a medical device is to ensure the product is safe, effective, and user-friendly.

A well-designed device should not only minimize the chances of user errors but also enhance the likelihood that any errors will be detected and corrected if they do occur. Systematic application of usability engineering principles, supported by user testing, is an effective approach to identifying and addressing design issues.

### 2.1. Formative Assessment

This study used formative assessment to evaluate the True Dose kit's usability during development. The focus was on understanding participant interaction with the kit's instructions and practical use, aiming to identify areas for improving instructional design and user experience. Insights were gathered from semistructured interviews and free-text responses in the Test Feedback Questionnaire (TFQ), shaping subsequent iterations of the kit to better meet user needs.

### 2.2. Summative Assessment

The summative assessment evaluated the overall effectiveness of the True Dose kit, focusing on user performance in sample collection accuracy, ease of use, and confidence. The evaluation was based on System Usability Scale (SUS) scores and performance metrics like blood volume collection, absence of air bubbles, and self-sampling duration. This data was enhanced with qualitative feedback to provide a comprehensive view of the kit's usability in real-world settings.

## 3. Material and Methods

The study was conducted at various locations, including a laboratory at Karolinska Institutet in Solna, participants' homes, and workplaces, between June 7 and June 25, 2024. Ethical approval was obtained from the Swedish Ethical Review Authority (Number: 2024-02661-02). Participants received oral and written study information and provided written consent before beginning the study activities.

### 3.1. Participants

Participants were recruited through multiple channels, including word of mouth, email, and social media advertisements, ensuring a diverse sample. Convenience and snowball sampling methods were used to recruit at least 20 participants, with an equal distribution of males and females across different age groups. Eligibility criteria included being 18 years or older and having no prior experience with capillary self-sampling to ensure that the usability of the kit was evaluated by novice users. No explicit exclusion criteria regarding health status were applied.

### 3.2. Kit Description and Handling

True Dose kits were supplied by True Dose AB (Solna, Sweden). Each kit contained microtubes prefilled with a precipitation solvent (isopropanol:methanol, 1:1, with 0.1% formic acid), and the caps were preloaded with 1 *μ*M IS and an additive solution on a polypropylene matrix. Upon addition of capillary blood, the IS was immediately released into the microtube's extraction solvent. Participants added 50 *μ*L of whole blood to the tube, sealed it, and manually shook it for 30 s to ensure thorough mixing.

While the primary aim of this study was to evaluate the usability of the True Dose kit, rather than conducting laboratory analyses, it is important to note that each 50 *μ*L blood sample is diluted directly in 1 mL of precipitation solvent, resulting in a 1:20 dilution factor. Validation data from ongoing work (submitted manuscript, Komninos et al., 2025) confirm that this dilution does not compromise detection sensitivity; matrix effects and background noise are reduced in parallel with the analyte concentration. The defined lower limit of quantification (LLOQ) and the simultaneous addition of the IS allow an accurate and robust method for liquid chromatography-tandem mass spectrometry (LC-MS/MS) analysis. In typical laboratory workflows, these prestabilized samples are centrifuged to remove precipitated proteins, and the supernatant is then directly injected into the LC-MS/MS system for quantitative TDM.

During the initial setup phase, we tested smaller-gauge lancets to assess whether they would be sufficient for capillary blood sampling in the True Dose kit. However, these smaller lancets did not provide an adequate blood flow to reliably fill the sample tubes. Consequently, we selected the Becton Dickinson blue lancets, which have a larger gauge and deeper penetration. This choice was based on our pilot observations, which showed that the larger lancets provided a more consistent and sufficient blood flow, essential for reliable self-sampling. Our approach aligns with recommendations in the capillary blood sampling literature, which suggest that penetration depths of up to 2.2 mm can ensure reliable blood flow [25]. The BD blue lancets feature a safety design that automatically retracts the blade after use, minimizing the risk of accidental sharp injuries and aligning with clinical practice guidelines [26].

### 3.3. Study Design

**Figure 1:**
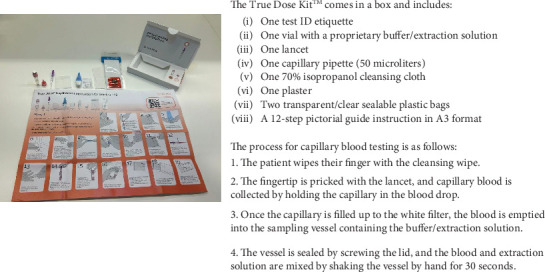
The True Dose kit and illustrated instructions for use.

Participants in this study were provided with a True Dose kit and asked to perform capillary blood self-sampling independently after watching a 7.26-min instructional video. The kit included all necessary materials and illustrated instructions in A3 format (Figure 1). During the self-sampling process, participants followed the written instructions while a researcher video-recorded them for subsequent analysis. Measures were taken to ensure that participants did not influence each other. No participants received assistance from anybody during the self-sampling process, as the sessions were designed to be performed independently.

In line with the standard procedure for capillary sampling in Sweden, participants were instructed to use warm water and perform pumping movements with their fingers before sampling to improve blood flow [26].

After completing the self-sampling, participants filled out a study form with demographic information. They then completed two questionnaires to evaluate the usability of the kit:

• SUS: A standardized questionnaire measuring the usability of the kit, with scores ranging from 0 to 100.
• TFQ: A study-specific survey to gather detailed feedback on participants' experiences, including free-text comments.

#### 3.3.1. Interview Procedure

After completing the questionnaires, participants were interviewed individually or in small groups, moderated by MC, between June 19 and June 29, 2024. They answered three open-ended questions about their experience with the True Dose kit, its ability to meet their needs, and suggestions for improvement. The same interview guide ensured consistency across formats, allowing each participant to express their views fully. Interview formats were adjusted to accommodate schedules while ensuring in-depth data collection.

The study initially planned focus group interviews with up to nine participants. However, due to scheduling conflicts with national holidays and summer vacations, the approach was adjusted, and interviews were conducted individually or in smaller groups to accommodate participants' schedules while ensuring thorough data collection.

### 3.4. Data Analysis

#### 3.4.1. User Performance

The primary outcome of this study was to evaluate users' performance during the capillary blood self-sampling process, which was assessed independently by two researchers (E.H. and M.C.). The analysis focused on three key performance metrics: (i) collection of sufficient blood volume: Success in collecting at least 50 *μ*L of blood, ensuring it reached the white filter, was assessed as “passed” or “failed” by both researchers; (ii) absence of air bubbles: The quality of the blood sample was evaluated by visually inspecting for air bubbles, with each sample rated “passed” or “failed” by each researcher independently; and (iii) duration of the self-sampling process: Each researcher recorded the total time it took to complete the self-sampling process—from kit opening to sample sealing—and calculated and reported the mean duration.

#### 3.4.2. Questionnaires

SUS is a 10-item questionnaire with a Likert scale designed to measure the usability of a system [21]. The SUS includes five positive and five negative statements. Scores range from 0 to 100, with 68 considered the average benchmark for usability and scores above 80 indicating high usability. Each SUS item was scored on a scale from 0 to 4, with adjustments made for positively and negatively worded items. The final SUS score was calculated by multiplying the adjusted scores by 2.5. Descriptive statistics, including mean, standard deviation, median, and interquartile range, were calculated for each SUS item and the observed performance metrics. The Swedish SUS has previously demonstrated high conformity with the original English version, high internal consistency (Cronbach's *α* = 0.852), and construct validity, indicating that the scale is psychometrically robust in a Swedish context [27]. No changes were made to the wording of the SUS items.

The TFQ is an 11-item questionnaire developed explicitly for the study to gather detailed feedback on participants' experiences with the capillary self-sampling kit [13]. The TFQ questions were based on existing usability and user experience literature. The selection of these items was based on their relevance to the context of self-sampling and usability of capillary blood at home and to enable a mixed-methods design that combined quantitative and qualitative feedback. The current study team adapted the items to ensure clarity and contextual appropriateness, but the specific items were not psychometrically validated prior to the study. The final questionnaire was reviewed internally to ensure comprehensibility and face validity.

### 3.5. Qualitative Data Analysis

The quantitative part consisted of both interviews and free-text comments in the TFQ.

All interviews were audio-recorded using a mobile phone and later transcribed verbatim. The interviews and the free text comments in the TFQ were analyzed using qualitative content analysis with an inductive approach, as described by Patton and Bogers et al. [28, 29]. Two researchers (M.C. and A.J.) independently conducted the coding. Codes with similar content were merged into themes. Each participant was assigned an individual number for identification. All authors participated in the final analysis.

### 3.6. Integration of Quantitative and Qualitative Data

**Figure 2:**
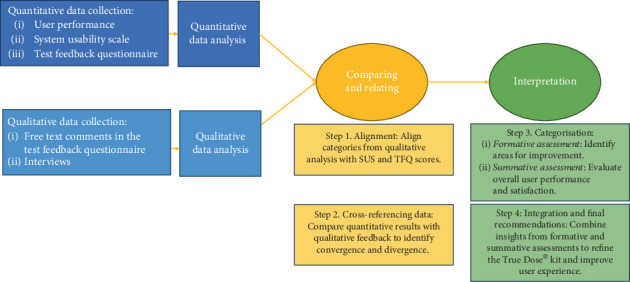
Convergent parallel design and mixed methods based on four steps.

Integrating findings from quantitative and qualitative phases followed a convergent parallel design [30] to comprehensively understand the user experience with the True Dose kit (Figure 2). The integration process was structured in four steps: (i) alignment: Qualitative categories were matched with corresponding quantitative data from the SUS and TFQ, such as aligning instructional design issues with SUS scores on complexity and ease of use; (ii) cross-referencing: Quantitative results were cross-referenced with qualitative feedback to identify convergence and divergence, comparing high SUS scores with comments on ease of use and lower scores with reported challenges; (iii) theme identification: Integrated data informed formative and summative assessments, focusing on areas for improvement and evaluating overall user performance and confidence; and (iv) illustration: Selected interview quotes highlighted critical categories in the quantitative analysis, such as aligning feedback on instructional complexity with SUS scores reflecting perceived complexity.

## 4. Results

Between June 19 and June 29, 2024, 25 participants were enrolled in the study.  Table 1 summarizes the demographic characteristics.

### 4.1. User Performance

Of 25 participants, 23 (92%) successfully filled the capillary to the white filter and collected the required 50 *μ*L of blood into the test tube, meeting the threshold for sufficient sample collection. Two participants had difficulty filling the capillary: one due to low blood flow and incorrect finger pricking and the other due to improper instrument use despite adequate blood flow. In 24 participants, the collected blood samples were free of air bubbles (Table 2).

The median time to complete the entire self-sampling process, from opening the kit to sealing the sample, was 10.42 min (IQR: 5.07 min). The median time from pricking the finger to inserting the capillary into the test tube was 1.48 min (IQR: 1.08 min). One participant encountered issues with the capillary Minivette POCT 50 *μ*L, which affected the sample (Table 2).

### 4.2. SUS Questionnaire

The overall SUS score for the capillary test system was 82.70 (SD = 13.227), indicating high usability (Table 3).

### 4.3. TFQ

The TFQ results indicate that participants generally disagreed with statements suggesting difficulties with the process, reflecting a more positive experience overall (Table 4).

### 4.4. Qualitative Phase and Integration of Quantitative and Qualitative Data

#### 4.4.1. Formative Assessment

The formative assessment revealed that while the True Dose kit was generally usable, user confidence and stress management challenges were evident. These challenges stemmed from the complexity of instructions and the manual operation of the lancet, which some participants found cumbersome and anxiety inducing. Quantitative data supported these findings, indicating that participants often felt overwhelmed by the detailed steps and experienced stress during the self-sampling process. For instance, despite a high usability score (SUS score of 82.70), clear feedback highlighted the need for more straightforward, visually guided instructions (Table 5).

#### 4.4.2. Summative Assessment

The summative assessment further underscored the connection between user confidence, stress, and procedural consistency. Ninety-two percent of participants collected the required blood sample, indicating a smooth process. Concerns about blood flow management and the adequacy of the sample contributed to variability in how participants followed the procedure, such as the proper shaking of the tube for 30 s. These findings highlight the need for clear, confidence-building feedback mechanisms during the process, which could reduce stress and promote a more consistent, reliable experience (Table 6).

## 5. Discussion

This study explored the usability of the True Dose kit for capillary blood self-sampling on a single occasion, through a comprehensive, mixed-methods approach. We intentionally included individuals from the general population without prior finger-prick experience to reflect typical end-users. Integrating qualitative and quantitative data provided a nuanced understanding of initial user performance and experience. Our findings indicate a high level of usability, with a mean SUS score of 82.7, suggesting that participants found the kit generally intuitive and straightforward to use. While the overall usability score was high, several critical areas for improvement were identified to enhance user experience and ensure consistent performance across diverse user groups.

A key observation was that 92% of participants successfully collected the required blood volume, with a median duration of the entire self-sampling process being 10.42 min (IQR: 5.07 min) and a median time from finger prick to filling the capillary tube of 1.48 min (IQR: 1.08 min). These findings align with prior studies investigating the feasibility of capillary blood self-sampling in nonclinical settings. For instance, Otten et al. reported that nearly all participants were able to collect adequate blood samples for TDM using finger-prick-based capillary sampling at home, even without prior training [31]. Similarly, Liu et al. demonstrated that self-sampling was feasible and generally well accepted by individuals with no prior experience with venipuncture, supporting the potential for broader implementation of such approaches in decentralized clinical trials [13].

Despite the high overall usability score, participants in our study expressed concerns regarding the complexity of the written instructions and reported feeling stressed during the sampling process. This was reflected in the qualitative data, which revealed that participants sought more concise, visually oriented guidance to reduce cognitive load. These findings are consistent with previous research emphasizing the importance of clear, intuitive instructions in facilitating self-sampling procedures. For example, Boffel et al. reported that visually guided instructions significantly improved blood microsampling by adolescents at home, even without prior experience [12]. Spooner et al. similarly highlighted the crucial role of user-friendly instructions in ensuring user confidence and compliance during self-sampling [9]. Lingervelder et al. found that simplified instructions boosted patient acceptance and performance of at-home blood collection devices [14]. Otten et al. also demonstrated that clear instructions contributed to successful capillary self-sampling for TDM [31]. These studies collectively underscore the need for well-designed, straightforward guidance materials to optimize user experience and ensure consistent, reliable self-sampling performance.

In this study, 23 out of 25 participants (92%) successfully collected the required blood volume (50 *μ*L), while 24 out of 25 samples (96%) were free of air bubbles. BD blue lancets were chosen after preliminary testing indicated they provided sufficient blood flow for reliable self-sampling. Although these larger-gauge lancets are known from previous studies to ensure more consistent blood volumes [32], we did not systematically evaluate user comfort or compare different lancet types. Consequently, while the choice of these lancets contributed to the high success rate of blood collection, our study does not offer specific insights into the relative comfort or usability of different lancet designs. Importantly, the proper disposal of these lancets and other used materials is also critical for compliance with local waste disposal regulations.

A key observation was that only 52% of participants adhered to the instruction to shake the tube for at least 30 s, highlighting variability in adherence to the protocol's final steps. Participants frequently expressed uncertainty about whether they had performed these steps correctly, such as whether the fluid had fully changed color or whether the shaking step was sufficient. This mirrors the results of Schröder et al., who noted that elderly or immunocompromised individuals also exhibited variability in self-sampling procedures at home, emphasizing the need for clearer guidance and real-time feedback to support consistent performance [33]. This variability in adherence underscores the importance of incorporating clearer, real-time feedback mechanisms and more intuitive instructional materials to support consistent user performance. Such refinements align with established usability engineering frameworks [34, 35], which emphasize reducing uncertainty and enhancing reliability in medical device operation.

The True Dose kit's technology, featuring immediate IS integration and direct protein precipitation, streamlines laboratory workflows by eliminating the need for plasma separation, drying, or manual stabilization steps [18, 36]. This prestabilized approach reduces operator variability and preanalytical errors, unlike traditional venous or DBS sampling [18, 36], supporting more reliable whole-blood TDM while reducing laboratory workload [10, 15, 20, 37].

Whole blood sampling provides enhanced reliability for drugs like cytotoxics that partition into red blood cells, as it minimizes plasma-related fluctuations and variations in protein binding [19, 36, 38]. The True Dose kit's liquid-based whole blood sampling design aligns with this advantage, providing a consistent and user-friendly solution for decentralized TDM [37]. Because the samples are liquid whole blood rather than dried, they must be handled as biohazardous material during transport and laboratory processing, following standard biosafety protocols.

This study has several limitations. First, the sample size was relatively small and based on convenience sampling, which may limit the generalizability of the findings. Second, usability was assessed on a single sampling occasion, and repeated usability over time, critical for long-term TDM in clinical practice, was not evaluated. Addressing these limitations in future studies, particularly with a focus on elderly populations and patients with chronic conditions, will be essential to confirm the broader applicability of the True Dose kit.

In conclusion, this study demonstrates that the True Dose kit has high usability potential for at-home capillary blood self-sampling, with performance metrics and user feedback comparable to other microsampling approaches in the literature. The identified challenges, particularly regarding instructional clarity and stress management, should be addressed in future iterations of the kit to optimize the user experience. These findings support the continued development and refinement of user-centered sampling technologies to enhance decentralized clinical trial feasibility and routine TDM in diverse patient populations.

## Figures and Tables

**Table 1:** Demographic characteristics.

**Demographic factor**	**Category**	**n** ** (%)**
Gender	Female	17 (68)

Age (years)	Median (IQR)	50.0 (36–58)
	Range	22–76
	Aged 65+	4 (16)

Education level	High school	6 (24)
	University/college	19 (76)

Medication status	No ongoing medication	14 (56)

Abbreviation: IQR, interquartile range.

**Table 2:** Performance metric.

	**Median (IQR)**	**n** ** (%)**
Whole test procedure duration of minutes (seconds)	10.42 (5.07)	—
Finger prick to capillary filling duration minutes (seconds)	1.48 (1.08)	—
Capillaries filled to the white filter	—	23 (92%)
Tube shaking for at least 30 s	—	13 (52%)

Abbreviation: IQR, interquartile range.

**Table 3:** Descriptive statistics for each item of System Usability Scale (SUS) questionnaire for True Dose kit.

**Item**	**Mean**	**(SD)**
1. I think I would like to use the system frequently.	2.76	1.234
2. I found the system unnecessarily complex.	3.68	0.690
3. I thought the system was easy to use.	3.04	0.935
4. I think that I would need the support of a technical person to be able to use the system.	3.88	0.440
5. I found that the various functions in the system were well integrated.	3.04	0.735
6. I thought there was too much inconsistency in the system.	3.32	0.988
7. Most people would learn to use the system very quickly.	3.24	0.779
8. I found the system very cumbersome to use.	3.28	0.792
9. I felt very confident using the system.	3.20	1.080
10. I needed to learn many things before I could get going with the system.	3.64	0.569
Total SUS score	82.70	13.227

**Table 4:** Test feedback questionnaire items.

**Questionnaire item**	**Mean**	**SD**
The finger prick device (lancet) was difficult to use	4.4	0.995
The collection tube was difficult to fill	3.6	1.635
The whole procedure was more complicated than expected	3.9	0.971
The process was more trouble than it was worth	4.2	0.866
The procedure caused stress	3.6	1.118
The written instructions were easy to follow and understand	1.6	0.810

*Note:* The interpretation of the scores is as follows: 1 = *strongly agree*; 2 = *agree somewhat*; 3 = *neutral*; 4 = *disagree somewhat*; 5 = *strongly disagree*.

**Table 5:** The integrated data were used to conduct formative assessments.

**Theme**	**Category**	**Quantitative reflection**	**Quote**
1: Instructional design challenges	1.1: Complexity of instructions	The complexity of the instructions was reflected in the SUS scores, where some participants rated the system as unnecessarily complex (SUS Item 2; Mean = 3.68; SD = 0.690). Additionally, participants indicated that they needed to learn many things before using the system effectively (SUS Item 10; Mean = 3.64; SD = 0.569). These scores suggest that the instructional design might have been overly complicated, requiring users to invest significant cognitive effort to understand and follow the steps.	“I think the instructions should be shortened with simple step-by-step processes, without unnecessary details like the plaster.” Participant 24
	1.2: Preference for visual guidance	The SUS score supported the preference for visual cues over textual instructions, indicating that participants found the system integrated but still somewhat inconsistent (SUS Item 5; Mean = 3.04; SD = 0.735; SUS Item 6; Mean = 3.32; SD = 0.988). The need for more intuitive and visually guided steps is evident from the slightly lower score for ease of use in the TFQ (TFQ ease of use; Mean = 4.4; SD = 0.995).	“I almost missed labeling the sample tube with the ID because it was not part of the main steps but mentioned separately.” Participant 9
2: Physical and emotional stress	2.1: Anxiety with manual lancet operation	The anxiety associated with manually operating the lancet is reflected in the relatively lower scores for feeling confident while using the system (SUS Item 9; Mean = 3.20; SD = 1.080). This suggests that some participants were uncertain or uneasy during the procedure, which could have been mitigated by a more user-friendly design.	“I was a bit skeptical about how the lancet worked... it would be easier if it had a clearer trigger mechanism.” Participant 18
	2.2: Blood flow management stress	Stress related to blood flow management was echoed in the TFQ, where the process was rated somewhat stressful (TFQ stress; Mean = 3.6; SD = 1.118). Additionally, the TFQ item measuring the perceived difficulty of the procedure showed that participants found it more challenging than expected (TFQ overall difficulty; Mean = 3.9; SD = 0.971).	“I felt stressed because there was much blood, more than I expected.” Participant 21

Abbreviations: SD, standard deviation; SUS, System Usability Scale; TFQ, Test Feedback Questionnaire.

**Table 6:** The integrated data were used to conduct summative assessments.

**Theme**	**Category**	**Quantitative reflection**	**Quote**
3: Overall user performance	3.1: Successful blood collection	The quantitative data revealed that 92% of participants collected the required blood sample successfully. However, there was a discrepancy in how effectively they managed this task—the time taken to complete the self-sampling process (Median = 10.42 min; IQR = 5.07 min) and the specific task of filling the capillary to the white filter (Median = 1.48 min, IQR = 1.08 min) varied across participants, indicating differences in user experience and performance.	“It was hard to get enough blood, and I was not sure how to handle the situation.” Participant 17
4: Need for confirmation and feedback	4.1: Uncertainty in sample adequacy	Only 52% of participants correctly shook the tube for at least 30 s, as required. This suggests a gap in understanding or consistency in following the procedure, which correlates with the qualitative feedback expressing uncertainty about whether the process was executed correctly. The variability in the TFQ scores related to the collection process (TFQ collection process, Mean = 3.6, SD = 1.635) further supports this finding.	“I was confused about whether the fluid was blue enough... it was unclear if I did it correctly.” Participant 9
	4.2: Execution consistency	The variability in execution is further underscored by the SUS scores reflecting inconsistency in the system (SUS Item 6; Mean = 3.32; SD = 0.988). These scores indicate that participants struggled with maintaining a consistent process, particularly in the final steps, leading to potential discrepancies in sample quality.	“I was not sure if I shook the tube long enough or if it was done right.” Participant 13

Abbreviations: IQR, interquartile range; SD, standard deviation; SUS, System Usability Scale; TFQ, Test Feedback Questionnaire.

## Data Availability

The data that support the findings of this study are available on request from the corresponding author. The data are not publicly available due to privacy or ethical restrictions.
